# A Case of Rupture of the Uterus

**Published:** 1879-10

**Authors:** P. W. Van Peyma


### A CASE OF RUPTURE OF THE UTERUS.

REPORTED BY P. W. VAN PEYMA, M. D.

Mrs. C., aged forty-four years, eighth pregnancy; previous confinements natural and easy; labor commenced 8 A. M., Sept. 7. A midwife was sent for, who soon arrived, and from whom and the husband the first part of the history was obtained. The pains gradually increased in force, until at about 3 P. M. they acquired an extraordinary power, when at 7 P. M. they suddenly ceased entirely, and the patient complaining that something was wrong, Dr. Hauenstein, of this city, an experienced obstetrician, was sent for. Dr. Hauenstein found the head well engaged, vertex presentation, right anterior position. Forceps were applied with great ease, and child delivered with slight effort. The child, to all appearances, had been dead for some time. The placenta was delivered in the usual manner, being found partially in the vagina; no hemorrhage; uterus contracted well. Immediately previous to delivery, the mother's pulse was found to be very frequent and feeble; she also appearing somewhat exsanguine. After the delivery of child and placenta, the pulse was noted at 140, and weak. Great tenderness of abdomen was observed, and repeated vomiting shortly took place. Morphia sulph. gr. ¼ injected hypodermically. Dr. Hauenstein remained with patient two hours after delivery, and left, feeling apprehensive as to the result.

Sept. 8, A. M.—Tenderness continuing, principally in left iliac region; pulse 144; temp. 101½; still vomiting; considerable tympanites; turpentine stupes applied; milk and turpentine injections; ice and whiskey by stomach; this, however, not retained; carbolic acid by vaginal injections. P. M.—Pulse 144; temp. 102; no material change; vomiting continuing; treatment continued; champagne substituted for whiskey; broths and beef-tea; tube passed into rectum; relieving tympanites slightly.

Sept. 9, A. M.—Pulse 135; temp. 102; passed a restless night. P. M.—Pulse 145; temp. 102; vomiting diminishing. From this time on until her death, at 7 A. M., Sept. 12, the pulse gradually increased, until, on the evening of the 11th, it reached 160; the temperature also increased slightly, but never exceeded 103, the point reached on the evening before mentioned. The patient during all this time, and up to within two or three hours of her death, was calm, hopeful, and perfectly rational. The night immediately preceding the day of her death, she slept a number of hours and awoke at 4 A. M., feeling very bright and entirely free from pain. She then soon became delirious, and remained so to the time of her death. The lochial discharge ceased within the first twenty-four hours. The autopsy was performed at 10 A. M., Sept. 14, by medical students, in the presence of Drs. Hauenstein, Diehl, Edw. Storck, Eugene Storck, Schade, Wetzel, and Van Peyma. In giving the history of the case to the physicians present, Dr. Hauenstein gave his diagnosis as rupture of the uterus, which, as will be seen, was verified by the post-mortem. The abdomen being opened, the peritoneum was found extensively inflamed; the left iliac fossa contained considerable extravasated blood. The rupture, which was on the left side and limited to the lower half of the uterus, extended nearly to the vagina, being at least two inches in length. A number of the physicians present satisfied themselves of its size and position by introducing the finger through it into the uterine cavity, the uterus being still in situ. The uterus on removal presented nothing else of interest.

